# Development and Application of Edible Coatings with *Malva sylvestris* L. Extract to Extend Shelf-Life of Small Loaf

**DOI:** 10.3390/foods11233831

**Published:** 2022-11-27

**Authors:** Iordanka Alexieva, Marianna Baeva, Aneta Popova, Hafize Fidan, Zhivka Goranova, Iliana Milkova-Tomova

**Affiliations:** 1 Department of Catering and Nutrition, Economics Faculty, University of Food Technologies, 4002 Plovdiv, Bulgaria; 2 Department of Tourism and Culinary Management, Economics Faculty, University of Food Technologies, 4002 Plovdiv, Bulgaria; 3 Institute of Food Preservation and Quality, Agricultural Academy, 4002 Plovdiv, Bulgaria

**Keywords:** antimicrobial activity, moisture loss, firming kinetics, microbiological characteristics, freshness, shelf life, mallow, bakery products

## Abstract

Edible coatings that have a recognized ecological effect are an alternative to retard the processes of moisture evaporation and mold growth in bakery products. The aim of the present research was to study the influence of *Malva sylvestris* L. (mallow) flowers’ extract on the antimicrobial activity of edible coatings of three types of polysaccharides, pectin/P/, xanthan/X/ and carboxymethylcellulose/C/, as well as to analyze their effect on the freshness and microbiological status of coated small loaves during storage. It was found that the presence of a mallow extract had a positive effect on the fungicidal and yeasticidal activities of the X and C coatings. The loaves were evaluated for their physical (moisture, color), textural (firmness and crumb firming kinetics) and microbiological characteristics. The coatings (P, X and C) with mallow extract had the strongest moisture-retaining effect on the loaves’ crumb. The coatings with X and with P (with/without mallow extract) significantly slowed down the crumb firming process, and the value of the rate constant for the crumb firming (k) is the lowest for the X coating—0.1815 day^−1^. The smallest changes in the crust color were reported when mallow-based coatings were used. They have also been proven to have the lowest microbial load when they are stored for up to three days. This study shows that polysaccharide edible coatings with an active mallow component have significant potential to extend the shelf life of bakery products.

## 1. Introduction

One of the major factors determining the quality and safety of bakery products is water and its state [1]. The processes of water diffusion and evaporation, unwanted structural–mechanical and organoleptic changes, as well as the sanitary-hygienic status deterioration of the bakery products occur during storage [2]. It is known that the structural–mechanical and colloidal properties of the crumbs change during storage, and this originates from the complex redistribution of moisture between high molecular substances (mainly starch and proteins), resulting in visible crumb staling [3].

State-of-the-art food preservation is no longer focusing on simple preservation, but on sustainability and health enhancement, as well as environmental friendliness [4]. Edible films and coatings are recognized as an alternative to retard the processes of moisture evaporation and mold growth in bakery products. [5,6]. When they are applied to the food product, they become part of it, and they can be absorbed when they are consumed, which among other advantages, has also a marked ecological effect [7]. Packaging with edible coatings or films blocks the migration of food components (enzymes, sugars, salts, etc.), protects the products from oxidation, and prevents the diffusion of moisture and aromatics [8]. Edible coatings create additional mechanical protection, color and gloss for the products [9].

Edible films and coatings are defined as a widely interacting network that possesses a three-dimensional structure. They are developed from polymers of natural origin, most often polysaccharides, proteins and lipids [10]. Plasticizers and surfactants can be added to these polymers in order to increase the food coating’s functionality. The water-holding capacity of the polysaccharides (pectin, starch, xanthan, carboxymethylcellulose, alginates, carrageenan, chitosan, etc.) makes them a common component of the edible films and coatings used to extend the shelf life of foods [11]. Pectin is considered to be widely applicable in edible films and coatings due to its diverse properties [12]. Pectin’s hydrocolloid and polyelectrolyte properties determine its unique abilities: to firmly retain the water in colloidal systems by stabilizing them, to be easily plasticized with glycerol due to its hydrophobic groups by adsorbing lipoid organic substances and its pronounced cation exchange ability, which forms its health protective properties. Bartolozzo et al. [13] prove that triticale flour-based coatings preserve the freshness of muffins by retarding the crumb firming process. Ferreira Saraiva et al. [14] investigated the effect of active potato starch edible films on the shelf life of mini panettone. The results showed that the two edible films, with/without an active ingredient, had a preserving effect on the mini panettone in terms of its texture, moisture content and microbial load. In a number of studies on bakery product staling, the effects of multicomponent edible and biodegradable coatings and films obtained by combining different polysaccharides, proteins and lipids have been examined in order to take advantage of the characteristics and properties of each compound, as well as to achieve a synergistic effect between them [15,16,17]. Whey protein and pectin edible coatings in the presence of transglutaminase have also been developed to be used as a barrier against moisture transfer and to improve the microbiological quality of biscuits [18]. The developed coatings have revealed their potency in maintaining the biscuits’ textural characteristics for up to 50 days after baking, and this is of particular importance for the consumer’s evaluation of this type of product. In another study [19], it was found that polysaccharide–lipid coatings applied on dry bakery products reduced the moisture absorption in crackers during storage. Bravin et al. [19] demonstrated that the inclusion of lipids in the composition of the edible coatings can improve the protection of dry bakery products against moisture absorption.

The edible coatings’ matrices can easily include antioxidant and antimicrobial components to help extend their shelf life [20]. The introduction of antimicrobial agents into edible coatings offers a number of advantages in comparison to those of the traditional preservatives: direct application to the surface of the food without penetrating the food matrix, and they still precisely exhibit their activity [20]. Antimicrobial edible coatings allow the preservation of the food’s quality, eliminating or at least limiting the development of foodborne pathogenic microorganisms (*Listeria moncytogenes*, *Salmonella* and *E. coli*) [21]. Many antimicrobials are incorporated at concentrations of 0.1–5% *w/w* of the packaging material, especially in the case of edible coatings [22]. In order to make the correct choice of the most suitable antimicrobial agents, it is necessary to know the basics of their impact on the target microorganisms. The microbiological quality of the bakery products is related to the development of the microflora that are characteristic of these products, mainly molds and yeasts, which are the main causes of microbiological spoilage. Less commonly, the spoilage is caused by bacteria, as this is due to reduced water activity in some products and also to a reduced pH [23]. A number of researchers found that edible coatings with bee wax, oils from various seeds, herbs and spices, nano emulsions, as well as the preservative nisin, demonstrated increased efficacy on the molds’ growth in bread and sweet bakery products [24,25,26,27,28]. Coatings prepared with chitosan are also proven to have fungicidal activity [29]. Essential oils and other extracts of plants, herbs and spices can be food preservatives with great antimicrobial and antioxidant effects for the bakery products. Given their high concentration of terpenes, terpenoids and phenolic compounds (about 85%), the essential oils exhibit strong antimicrobial properties. Cinnamon oil (with active ingredient cinnamic aldehyde) and clove oil (with active ingredient eugenol) have been used in the development of polysaccharide-based edible coatings [30,31]. An extended shelf life of the microbiological quality of the analyzed bakery products with such coatings and their preservation for a longer period of time were found.

*Malva sylvestris* L. is a medicinal plant that is found in countries in Europe and Asia [32]. It has been reported to possess antimicrobial, antioxidant and anti-inflammatory activity, among others [33]. Authors have proven that the flowers are more active in comparison to the leaves [32,34]. The various biological activities of the plant can be attributed to its valuable compounds such as organic acids, vitamins, flavonoids and other heath-contributing compounds [35].

The aim of the present research is to study the influence of *Malva sylvestris* L. (mallow) flowers’ extract on the antimicrobial activity of edible coatings of three types of polysaccharides, pectin/P/, xanthan/X/ and carboxymethylcellulose/C/, as well as to analyze their effect on the freshness and microbiological status of small loaves during storage.

## 2. Materials and Methods

### 2.1. Materials

The apple pectin (P) was highly esterified at a degree of esterification of 63% (62% purity, 125,000 molecular mass). The carboxymethylcellulose (C) used in the study was a product of Noviant (Nijmegen, The Netherlands), which has the trade name Cekol^®^. Xanthan (X) was a product of bacterial crop *Xanthomonas campesteris* fermentation. The raw coconut oil was produced and packaged by “Dragon superfoods”, and it was purchased at a local “Lidl” store in Plovdiv, Bulgaria. Glycerol was purchased from a local market, and it was authorized by the Ministry of Health in Bulgaria. All of the reagents and chemicals were classified as “pure for analysis”. Distilled water was used. A 70% ethyl alcohol extract from the dried flowers of *M. sylvestris* was obtained in a manner that was described by Alexieva et al. [36]. The resulting extract was filtered and stored at 4 °C. The small loaf had a raw composition because of the raw material types that were used for the dough kneading—wheat flour type 500 (GoodMills, Bulgaria EAD, 0.5% ash content, 14% moisture basis), dry yeast (instant, Lesaffre Group, Lille, France), table salt, sunflower oil and water. Standard raw materials were used and were purchased from a local store in Plovdiv, Bulgaria, and they were approved by the Ministry of Health in Bulgaria.

### 2.2. Preparation of Coatings

**Figure 1:**
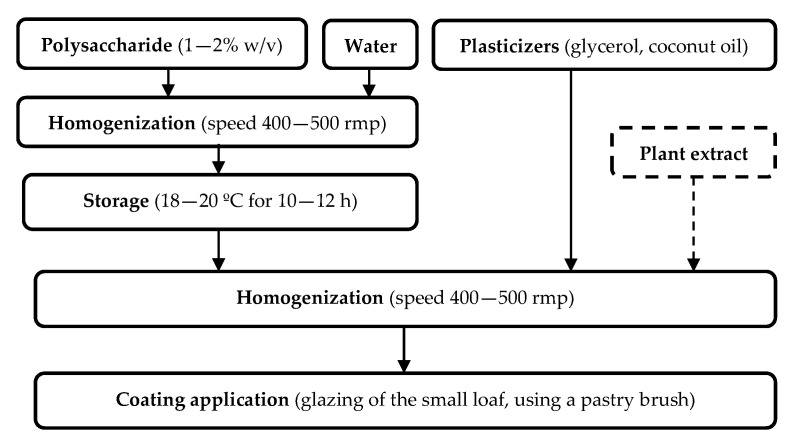
Technological scheme of food biopolymer coatings based on polysaccharides.

The recipe and technology of the multicomponent biopolymer coatings based on polysaccharides and coconut oil with/without a plant extract have been presented in previously published research [36]. The food biopolymer coatings’ preparation technology is shown in Figure 1.

A 1–2% *w*/*v* aqueous solution of each polysaccharide was used as a stabilizer in the edible coating which was prepared in the form of an emulsion. The coating hydrophobicity was increased by adding coconut oil to the emulsion. Glycerol was used as a plasticizer (0.5 mL/g biopolymer). The mallow (*M. sylvestris*) extract was directly incorporated into the emulsion coating, representing 8% of the emulsion coating.

Using the brushing method, the small loaves were glazed with the coatings using a pastry brush after cooling them for 2 h at room temperature (temperature of 20 ± 2 °C). Each loaf was covered with 10 mL of the appropriate edible coating on the top, side and bottom surfaces using a clean pastry brush to produce one layer.

### 2.3. Preparation of Small Loaf

The dough formulation of the small loaf was as follows (based on flour weight): 100% wheat flour, 60% water, 1.6% salt, 1.5% sunflower oil and 0.75% dry yeast. All of the dry ingredients were equilibrated at room temperature before their use. The water was pre-warmed to 30 °C and added at the beginning of the mixing step. A one-stage method was used to prepare the dough. The dough was made by mixing all of the ingredients in a mixer (machine Kemper Type SPL, Rietberg, Germany) at a medium speed for 10 min. It was fermented for 120 min at 30 °C. Later, the dough was formed manually, put into aluminum molds, and then, fermented for another 90 min. The small loaves were baked in aluminum molds each containing 60 g dough and placed in an electric continuous heating oven at 200 °C for 20 min. The small loaves were cooled to room temperature.

### 2.4. Antimicrobial Activity of Edible Coatings

#### 2.4.1. Test Microorganisms

Strains of foodborne pathogenic bacteria, supplied by the National Bank for Industrial Microorganisms and Cell Cultures, were used as test microorganisms. The antimicrobial activity was tested against the Gram-(+) bacteria—*Listeria monocytogenes* NCTC 11994 and *Staphylococcus aureus* ATCC 25093; the Gram-(−) bacteria—*Escherichia coli* ATCC 8739 and *Salmonella enterica subsp. enterica serovar Abony* NCTC 6017, a yeast strain of *Candida albicans* ATCC 1023; and a fungal strain—*Aspergillus brasiliensis* ATCC 16404. The selective media were Listeria Oxford Agar Base with an additive containing cycloheximide (Biolife), ENDO agar (Merck), LEIFSON Agar (Merck), Baird Parker Agar Base (Biolife) with a yolk tellurite supplement, Plate Count Agar (Merck) and Sabouraud Dextrose Agar (Merck), respectively.

#### 2.4.2. Determination of the Antimicrobial Activity of Edible Coatings

The determination of the antimicrobial activity of the edible coatings was made according to Atwaa et al. [37].

### 2.5. Storage of Small Loaves and Shelf-Life Analysis

The small loaves were stored under standard conditions (temperature of 20 ± 2 °C and relative humidity up to 75%) for three days including the production date, which is in agreement with the requirements for storage of packed bread according to the Bulgarian State Standard 3450-84 [38,39]. The control sample was an uncoated small loaf. Three batches of each type of small loaf were analyzed.

#### 2.5.1. Crumb Moisture Content and Moisture Loss

The moisture content of the crumb stored up to 3 days was measured according to the procedure described in AACC method 44-15.02. [40]. The determinations were carried out in triplicate. Moisture loss (%) was calculated by subtracting the moisture content of the loaf crumb during storage from the initial moisture content.

#### 2.5.2. Texture Analysis of Loaf Crumb

The analysis of the texture of the crumb stored for up to 3 days was performed. The firmness of the loaf crumb was measured by a texture analyzer (FT 327 pressure tester, TR Turoni, Alfonsine, Italy) using the stainless-steel plunger of 11 mm diameter which was mounted on a 13 kg maximum compression head. The pressure to penetrate was expressed in kg/cm^2^. The penetration distance was 20 mm (50% of the sample height).

#### 2.5.3. Firming Kinetics of Crumb Loaf Using the Avrami Model

A modified Avrami equation was applied to study the changes in the crumb firmness along time, according to Armero and Collar [41]. The Avrami equation (Equation (1)) considered that the fraction of the total change in the crumb firmness (θ) was a decreasing exponential function with respect to time. The mathematical parameters of the Avrami equation (Equation (1)) applied for loaf firmness (3 days) were based on starch retrogradation:

θ(t) = (F_max_ − F_t_)/(F_max_ − F_0_) = exp (−k.t^n^) (1)

where θ(t) was the non-firmed fraction of crumb loaf at time t; F_0_, F_t_, and F_max_ were the firmness values at 0 time (two hours after baking), t time, and infinite time (max time—3 days), respectively; k was the constant rate of the firming process (with units of day^−1^); n was the Avrami exponent (indicative of the nucleation type and crystal growth geometry) [42]. Normally, the important rate constant k is given as the time constant, 1/k, which describes the loaf crumb firming rate (i.e., the higher number was, then the slower the firming process was). All of the parameters were obtained from the modeling process. The restricted Avrami equation considering *n* = 1.0 was also applied.

#### 2.5.4. Color Analysis

A PCE-CSM 2 (PCE-CSM instruments, Germany) with a measuring aperture of 8 mm was used to determine the color parameters of the loaves’ crumbs and crusts. The estimated parameters were L* (lightness—ranging from 0 (black) to 100 (white)), a (representing the red–green opponent colors), b* (representing the blue–yellow opponent colors), C* (chroma—color saturation), and h* (hue angle—color tone). The total color difference (∆E) between the control and the sample of the small loaf coating was calculated according to the CIE76 color difference equation [43,44].

#### 2.5.5. Microbiological Analysis

The microbiological analysis of the loaves was carried out according to the Bulgarian State Standard 3412-79 [38]. Analyses for the total plate count [45], molds and yeasts [46], coliforms [47], *Salmonella* species [48] and coagulase-positive staphylococci [49] were also conducted. The total plate count (TPC) in 1 g of loaf sample, the total number of molds and yeasts in 1 g of loaf sample, the coliforms in 1 g of loaf sample, *Salmonella* species in 25 g of the loaf sample and coagulase-positive staphylococci in 1 g of loaf sample were determined.

### 2.6. Statistical Analysis

A relevant data analysis was performed with the use of MS Excel software. The results are presented as mean ± SD (standard deviation), which were formed from at least 3 repetitions of the assays. The one-way ANOVA and a Tukey–Kramer post hoc test (*p* ≤ 0.05), which were conducted as described by Assaad et al. [50], aided in the statistical analysis. The Pearson’s correlation coefficients between the moisture content and firmness were plotted with the use of Excel STAT Cloud function (MS Excel 365).

## 3. Results and Discussion

### 3.1. Antimicrobial Effect of Edible Coatings

**Figure 2:**
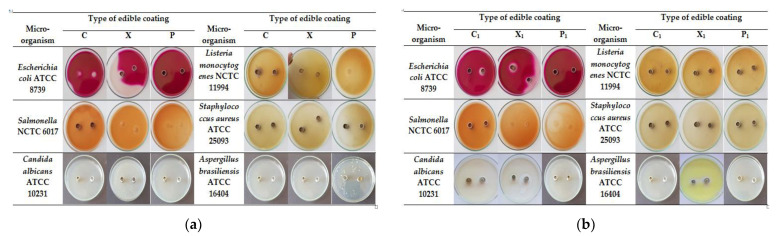
Images of the zones of growth inhibition of microorganisms (mm) in selective media for 0.10 mL and 0.15 mL of different types of edible coatings (at the 48th hour): (**a**) without mallow extract; (**b**) with mallow extract; P—pectin; C—CMC; X—xanthan, index 1 indicates coatings in which mallow extract (*M. sylvestris* L.) has been added.

Table 1 and Figure 2 present the results regarding the zones of inhibition of the growth of the microorganisms (pathogenic bacteria, yeast and mold) in selective food media from different samples of edible coatings with/without the participation of a plant extract of common mallow (*Malva sylvestris* L.).

The edible coatings did not exhibit antimicrobial activity against the pathogenic microorganism *Staphylococcus aureus* ATCC 25093 in all of the tested samples. The edible coating P shows the strongest inhibitory effect against *Listeria monocytogenes* NCTC 11994 as the largest area of suppression was obtained at an exposure period of 48 h and at concentrations of the edible coating of 0.15 mL. The edible coating X shows the strongest inhibitory effect against *Salmonella enterica* NCTC 6017 and *Listeria monocytogenes* NCTC 11994 as the largest area of suppression was obtained after an exposure period of 48 h. The coating C exhibits the strongest inhibitory effect against *Escherichia coli* ATCC 8739 as the largest suppression zone was obtained after an exposure period of 48 h. From the investigated edible coatings with the participation of a mallow extract, the coating P_1_ is characterized by the most pronounced antibacterial activity, with an inhibition zone of 36 mm/0.15 mL against *Salmonella enterica* NCTC 6017. The strain of *Escherichia coli* ATCC 8739 is most sensitive to the edible coatings with the participation of the plant extract. The coating X_1_ (16 mm/0.15 mL and 11 mm/0.10 mL) is characterized by the most pronounced antibacterial activity against the pathogen, which is followed by the coating C_1_ (15 mm/0.15 mL).

The obtained results indicate that the presence of mallow extract has a positive effect on the fungicidal activity of the xanthan edible coating (X_1_) by increasing the zone of inhibition to 15 mm/0.15 mL against *Aspergillus brasiliensis* ATCC 16404. The coatings X_1_ and C_1_ exhibited yeasticidal activity against *Candida albicans* ATCC 10231 with a zone of inhibition at 17 mm/0.15 mL and 13 mm/0.15 mL, respectively. The edible coatings without mallow extract are not characterized by a suppressive effect on the tested molds and yeasts.

It is well known that bacterial and fungal growth can reduce food quality through discoloration, textural changes and the development of bad flavors. Therefore, the effectiveness of edible films and coatings in inhibiting bacteria, molds and fungi has been studied by several researchers. For example, Wang et al. [51] studied the antibacterial activity of edible coatings based on whey protein isolate nanofibrils and carvacrol against two Gram-positive bacteria strains (*Listeria monocytogenes* and *Staphylococcus aureus*) and two Gram-negative bacteria strains (*Salmonella enteritidis* and *Escherichia coli*). They reported no inhibitory activity against the four microorganisms, meaning that there were no inhibitory effects on them for the absence of an antimicrobial compound in the film. The inclusion of carvacrol increased the antimicrobial activity of the films nearly twice because the presence of carvacrol significantly strengthened the antimicrobial activity of the edible film. In another study [52], the authors reported that the use of chitosan edible coating containing cinnamon oil is a promising approach for the preservation of fresh-cut potatoes. The cinnamon oil exhibited the lowest microbial inhibitory concentration for four foodborne pathogens (*Listeria monocytogenes, Salmonella* Typhimurium*, Staphylococcus aureus* and *Escherichia coli* O157:H7).

This undoubtedly proves that plant extracts are suitable components in edible coatings in order to extend different foods’ shelf lives.

### 3.2. Effect of Edible Coatings on Food Properties (i.e., Texture, Crumb Moisture, Moisture Loss, Colour and Microbial Growth)

#### 3.2.1. Crumb Moisture and Crumb Firmness

**Figure 3:**
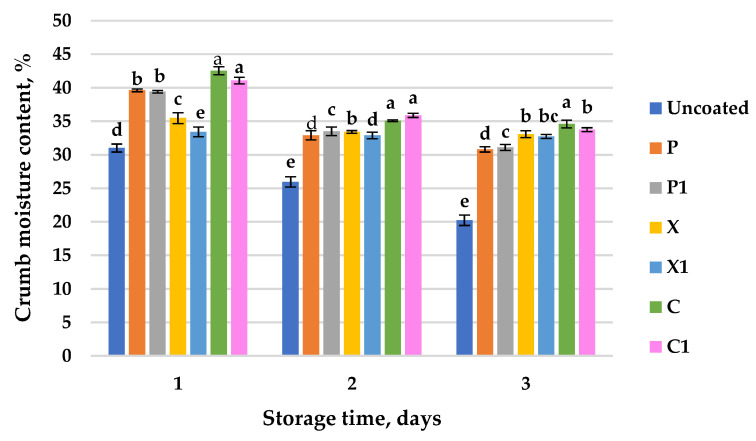
Changes in crumb moisture content of uncoated (control) and coated small loafs during storage (20 °C) for three days: P—pectin; C—CMC; X—xanthan, index 1 indicates coatings in which mallow extract (*M. sylvestris*) has been added. Values followed by the same letter for the same storage time interval are not significantly different (*p* ≤ 0.05, Tukey’s test).

The crumb moisture content of small loaves with/without a coating was observed for 3 days (Figure 3).

**Figure 4:**
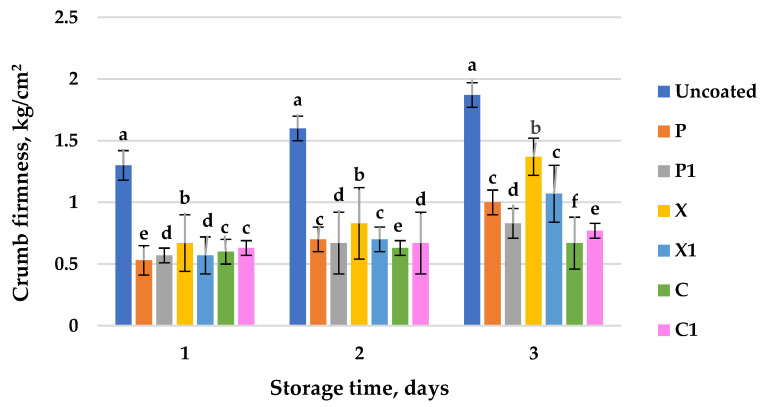
Changes in crumb firmness of uncoated (control) and coated small loafs during storage (20 °C) for three days: P—pectin; C—CMC; X—xanthan, index 1 indicates coatings in which mallow extract (*M. sylvestris*) has been added. Values followed by the same letter for the same storage time interval are not significantly different (*p* ≤ 0.05, Tukey’s test).

It can be seen that the loaves with the six types of coatings have a significantly higher crumb moisture content in comparison to that of the control. The crumb moisture content of the coated loaves varied in the ranges from 35.47 ± 0.81% to 42.53 ± 0.59% for the coatings without the mallow extract and from 33.40 ± 0.72% to 41.07 ± 0.50% for the coatings with the mallow extract, respectively, on the first day of storage, while the uncoated loaf showed a moisture value of 31.00 ± 0.60% for the crumb. No significant differences were found for this indicator, while statistical indistinguishability was proven at *p* ≤ 0.05 when the mallow extract was added to the basic edible coatings with C and P, respectively. A decrease in the crumb moisture of all of the examined small loaves was observed during the storage stage for up to three days. The change in crumb moisture during the storage stage was most significant for the control. The uncoated loaf (control) lost 10.78% of its moisture during the 3 days of storage. This proves that in the control sample, the rates of moisture change are more dynamic, and a more intense moisture loss leads to the faster firming of the loaf’s crumb (Figure 3 and Figure 4). When the mallow extract was added to the three types of base coatings, lower moisture losses in the loaf’s crumb occurred during storage. The loaves coated with the mallow extract lost an average of 5.44% of their moisture during the three days of storage, which was 5.34% less than that which was lost the control sample and 0.95% less than that which was lost in the coated samples without mallow extract. Therefore, it can be claimed that the P_1_, C_1_ and X_1_ coatings have the strongest moisture-retaining effect on the small loaves’ crumb during storage. During the three days of storage, in the loaves with the six types of multi-component edible coatings (with/without the mallow plant extract), the crumb moisture losses are the smallest in the loaves with coatings containing xanthan: the values reach 2.40% in the sample without the extract and 0.67% in the sample with the mallow extract (Figure 3). It can be concluded that all of the six multi-component edible coatings are effective in protecting against moisture loss. Similar results have been obtained in other studies which tested the application of starch gum-based multi-component edible coatings on rice cakes [28], pectin, alginate and whey protein on mini buns [15], as well as zein-based edible coatings on wheat bread [16] which led to a reduction in the moisture loss of the coated bakery products during the storage stage. Carboxymethyl cellulose as a component of edible coatings provides a barrier to moisture transfer, helping to maintain freshness [53], which is very well supported in the present results. The influence on the three hydrocolloids shows that carboxymethyl cellulose appears to be more suitable when it is compared to pectin and xanthan when one is considering moisture retention in the coated products.

The supplementation of hydrocolloids in order to test the physical properties of coated breads is not new to the research community [54]. Usually, all of the variations used lead to a reduced moisture loss. A different research study has point out a different hydrocolloid as being the most suitable one, thus, properties such as visual appearance, additional flavor and overall acceptability should be taken into account.

The effectiveness of the edible coatings with/without the mallow extract in controlling the small loaves’ staling and preserving their freshness, respectively, can be evaluated by measuring the texture parameters. Texture is one of the main characteristics of the bakery products. The loss of desirable texture results in shorter shelf life. Crumb firming is a preferred parameter used to evaluate the degree of staling. This study reflects the changes in the samples’ firmness (control and small loaves with six types of edible coatings) that occurred during storage. Changes in the crumb’s firmness during storage are shown in Figure 4. The firmness values of all of the samples increased during storage, with the most significant increase being in the firmness of the control (the loaf without coating), which was probably due to it having the greatest moisture losses (as seen in Figure 3). The loaves prepared with edible coatings containing the mallow plant extract are distinguished by the smallest crumb firmness in comparison to the control, and this regularity is manifested throughout the entire storage period. Small loaves coated without the mallow extract on the third day of their storage have an average of 45.81% less firmness (0.67 ± 0.21; 1.00 ± 0.10; 1.37 ± 0.15 kg/cm^2^), and in the samples containing the mallow extract, they have a 54.01% (0.77 ± 0.06; 0.83 ± 0.12; 1.07 ± 0.23 kg/cm^2^) lower crumb firmness in comparison to that of the control (1.87 ± 0.55 kg/cm^2^). The six types of edible coatings were found to be effective in producing softer loaves than the control was during storage. Similar trends were observed for the hardness of rice cakes coated with mung bean starch/guar gum-based edible emulsion coatings at the beginning of their storage [25]. Furthermore, coated rice cakes with a mixed starch-gellan gum coating showed lower hardness values than the uncoated product did after 4 days of storage at room temperature and 45% relative humidity [28]. Nallan Chakravartula et al. [17] reported that a pectin, alginate and whey protein monolayer coating reduced the hardness of mini buns by 22.20% in comparison to control samples. They explained that the hardness of the mini buns was negatively correlated with the moisture data. An analogous dependence has been presented in similar studies [15]. Le-Bail et al. [3] found a close relationship between crumb moisture loss and bread staling by studying the kinetics of amylopectin crystal formation, and consequently, crumb hardening. The current results also showed that the crumb moisture content of the loaves decreased during storage, while the firmness increased in parallel. The established inverse proportional relationship between the two parameters, expressed mathematically, has a negative correlation coefficient. Table 2 shows the Pearson’s correlation values between the moisture content and the firmness of the crumb of the small loaves studied in the process of storage for up to three days. All of the variables showed a negative relationship, and the samples were considered to be correlated if the absolute value correlation coefficients were >0.75. An exception is the loaf with the coating C_1_ on the third day of storage (r = −0.658). The variables associated with an ideal inverse relationship, with a coefficient equal to −1.000, were the firmness and moisture of the crumb of the coated loaf P on the third day of its storage, and of the coated loaf C on the first day of its storage.

The rate of the firming or hardness of the loaves (control and coated) can be quantitatively analyzed by using Avrami’s equation. The Avrami equation was originally developed to describe the crystallization of high molecular weight polymers, and it has been widely used for the kinetic analysis of starchy foods staling [3,49]. The Avrami exponent (*n*) depends on the nucleation mechanism and growth (*n* = 1–4). According to previous studies, starch crystallization, or the staling process, is caused by the linear growth of crystals due to instantaneous nucleation [50]. Thus, the firmness data were modeled by the Avrami equation, which is constrained by a fixed value for the Avrami exponent *n* = 1, thus giving good accuracy (Table 3). When *n* = 1, the constant rate of the firming process (*k*) can be determined from the slope of the graph with ln(Fmax – Ft), where time t is the axis.

As shown in Table 3, the loaf treated with the coating X has the lowest *k* value (0.1815 day^−1^), while the control sample has the highest *k* value (0.5280 day^−1^). Analogous results were presented Bartolozzo et al. [13], who reported that the treatment with an edible triticale-based coating decreased the rate constant for the crumb hardening (*k*) of muffins by 24% in comparison to that of the uncoated muffins. According to Armero et al. [41], a decrease in the value of *k* corresponds to slower crumb firming kinetics. The developed coatings X and X_1_ reduced the rate of the crumb firming of the small loaf by 62.20%, the coatings C and C_1_ reduced it by 14.64%, while the coatings P and P_1_ reduced it by 38.48% in comparison to the control. This proves that the experimental samples prepared with the six food coatings became stale slowlier than the control ones did, and they retained their freshness for a longer time. The crumb of the loaf without the coating will reach its final firming value faster than the samples with the six types of coatings will, which is evident from the time constant (1.89 days). The time constant represents the time for any given fraction of material to be converted into a stale form [55]. The larger the time constant is, then the slower the firming kinetics are. The crumb of the coated loaf X will reach its final firming value the slower than the others will (5.51 days). The coatings with X and with P (without and with mallow extract) significantly slow down the firming of the loaf crumb, which is evident from their time constants, and this will lead to the preservation of their freshness and softness for a longer period of time.

#### 3.2.2. Color

Color is one of the most important appearance characteristics as it directly affects the consumer’s preference for the food product. During storage, the moisture content of the small loaves decreased, which also affected their crust color. Discoloration occurs during storage, and it is often recognized by consumers as a sign of poor quality. Therefore, the influence of multi-component biopolymer coatings with/without a mallow extract on the qualitative color characteristics of bakery products during storage was tracked. The crust color parameters obtained for each small loaf are presented in Table 4.

**Figure 5:**
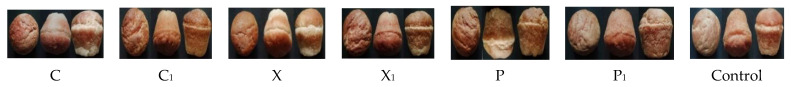
Photography images of small loaves at first day: Control—uncoated loaf; Loaf coated with: P—pectin; C—CMC; X—xanthan; index 1 indicates coatings in which mallow extract (*M. sylvestris*) has been added.

The obtained results indicate that the color parameters of the loaf’s crust change significantly when we applied biopolymer coatings without and with an extract of the mallow plant. The brightness L* of the crust changed the most, and for all of the samples with coatings, it is smaller than that of the control (on the first day of storage) as can be seen from the pictures in Figure 5. As it has been mentioned in other research, the observed color changes are most likely referenced to the Maillard reaction [56].

During storage, this dependence changed in the opposite direction. For the crust of the uncoated loaf, when it was stored for up to 3 days, the L* indicator decreased most significantly, and the red color indicator a* increased most significantly, and the metric angle of the color tone h* for the crust of this loaf reached their smallest values on the third day. The same trend is observed in the paper of Sopiwnyk et al. [57]. The crusts of the loaves with coatings have higher values of L* after they were stored for three days (from 50.83 ± 5.47 to 56.56 ± 1.61) in comparison to that of the control (49.22 ± 1.67), which showed a significantly brighter color than the control did. For the coating with pectin containing the mallow plant extract, the crust color change ∆E was the least extensive at 0.43, which is relative to the control on the first day of storage. During the storage process, the smallest changes in the crust color were recorded when food coatings containing the plant extract of forest mallow were used, with the ∆E varying between 1 and 3 days of storage for these coatings from 1.47 to 1.85. The largest change in the crust color ∆E between 1 and 3 days of storage was reported for the control—6.34. The crusts of all of the loaves, as expected, are darker than the crumbs of the loaves. The color parameters L*, a* and b* in the crumb of the loaves did not change significantly when the biopolymer coatings (with/without the mallow extract) were applied to them (Table 5). The same dependence is preserved when they were stored for up to three days.

#### 3.2.3. Sanitary Hygienic Status

The sanitary hygienic status of the studied loaves during their storage is presented in Table 6. The results for the total number of mesophilic aerobic and facultative anaerobic microorganisms, molds and yeasts confirms the low microbial load of the samples with edible coatings during storage. On the day of production, the presence of *Coliforms*, *Salmonella* and coagulase-positive staphylococci was not detected in the samples, which confirms the absence of additional contamination of the samples after their production. The same results were found throughout the entire storage period. According to these indicators, the studied small loaves are in accordance with BDS 8726-86 [38].

The increase in the microbial load and the presence of molds and yeasts during storage are most likely due to additional contamination from the air environment during storage (Table 6). The control sample was marked with the highest values. During the storage process, small amounts of yeast and mold were registered in the samples with the coatings X and C. Molds and yeasts were not detected throughout the entire storage period in the samples with coatings containing the mallow extract. *Malva sylvestris* L. clearly acts as an antimicrobial agent in the coatings. The test result for the total number of mesophilic aerobic and facultative anaerobic microorganisms and molds and yeasts confirmed the low microbial load of the coating samples during storage. The sanitary hygienic status of the loaves in the process of storage for up to three days is best when coatings with the addition of mallow extract were used.

Some studies have also demonstrated that *Malva sylvestris* L. extracts have antibacterial activity, and involving coatings with *Malva sylvestris* L. extracts has reduced the microbial growth on various food products [58]. To the best of our knowledge, *Malva sylvestris* L. extract was incorporated to the formulation of an edible coating for first time. The influence of various edible coatings with antimicrobial agents on the shelf life of food products were previously reported in the literature. For example, Bashir et al. [59] documented a reduction of the microbial population of the coated samples of chicken nuggets during storage with the increase of the coating solution. According to their findings, the total aerobic count was affected significantly by the coating formulations and the storage time. On the other hand, the coliforms population increased significantly in the uncoated samples in comparison to the coated chicken nuggets. The chitosan edible coating containing 0.2% cinnamon oil effectively maintained the quality of fresh-cut potatoes including inhibiting the browning, preventing the weight loss and maintaining their firmness. The reported results showed a decline in the total plate counts, yeast and mold counts, total coliform counts, the lactic acid bacteria count and *Listeria monocytogenes* in the edible coatings containing 0.2% cinnamon oil [52]. In another study, He et al. [60] determined the antimicrobial activity of kappa-carrageenan coatings enriched with cinnamon essential oil in pork meat in their work, and they reported that the coatings were able to delay the spoilage of pork meat for at least 2 days in comparison to that of the control. The effect of the xanthan coating containing various concentrations (0, 1, 2%; *w*/*v*) of the ethanolic extract of propolis was studied regarding the microbial indices in mackerel fillets stored at 2 °C for 20 days by Sheikha et al. [61]. The samples treated with xanthan coatings containing 1 and 2% of ethanolic extract of propolis were shown to have the maximum level of microbial inhibition (*p* < 0.05) in comparison to the uncoated samples (control) over the storage period.

## 4. Conclusions

The presence of mallow extract exhibited a positive effect on the fungicidal (xanthan) and yeasticidal (xanthan and carboxymethylcellulose) activity of the studied coating. The pectin coating with added mallow extract demonstrated an antibacterial activity against Salmonella NCTC 6017. All of the coatings with added mallow extract (P_1_, X_1_ and C_1_) had the strongest moisture-retaining effect on the loaves’ crumb during storage. The moisture losses in the crumb are the smallest for the loaf with the xanthan coatings: the values reached 2.40% in the sample without the extract and 0.67% in the mallow extract sample on the third day. The coatings with xanthan and pectin (without and with mallow extract) significantly slowed down the firming of the loaf’s crumb, and the rate constant for the crumb firming (k) is the lowest for the xanthan coating: 0.1815 day^−1^. In the process of storage, the smallest changes in the loaf’s crust color were recorded when we were using edible coatings containing mallow extract. The crumb color measurements showed similar values. The loaves with edible coatings, with the addition of the mallow extract, had the lowest microbial load after storage for up to three days. These studies prove that the use of polysaccharide edible coatings with an active component (mallow extract) are an effective factor for the shelf-life extension of bakery products as they preserve the small loaves’ freshness better during storage by reducing their moisture loss and decelerating the process of crumb firming as well as by allowing them to maintain an excellent sanitary hygienic status.

## Figures and Tables

**Table 1:** Zones of inhibition of the growth of microorganism (mm) in selective media of different types of edible coatings without and with plant extract (at the 48th hour).

Microorganism		Type of Edible Coating											
		P		P_1_		C		C_1_		X		X_1_	
		Concentration of Edible Coating, mL											
		0.15	0.10	0.15	0.10	0.15	0.10	0.15	0.10	0.15	0.10	0.15	0.10
*Listeria monocytogenes* NCTC 11994	Diameter * amimiof the zones, mm	19	*	*	*	*	*	*	*	12	10	*	*
*Staphylococcus aureus* ATCC 25093		*	*	*	*	*	*	*	*	*	*	*	*
*Escherichia coli* ATCC 8739		*	*	*	*	17	15	15	*	14	*	16	11
*Salmonella* NCTC 6017		*	*	36	10	12	*	*	*	14	11	*	*
*Candida albicans* ATCC 10231		*	*	*	*	*	*	13	*	*	*	17	14
*Aspergillus brasiliensis* ATCC 16404		*	*	*	*	*	*	*	*	*	*	15	12

P—pectin; C—CMC; X—xanthan; index 1 indicates coatings in which mallow extract (*M. sylvestris*) has been added. * Diameter of the zone equal to the diameter of the metal ring (6 mm) is considered to be a negative result.

**Table 2:** Pearson’s correlation coefficients of crumb moisture and firmness parameters of uncoated (control) and coated small loafs during storage for three days *.

Parameters	Crumb Moisture Content		
	1 Day	2 Days	3 Days
**Control**			
Firmness	−0.961	−0.999	−0.756
**P**			
Firmness	−0.8663	−0.870	−1
**P_1_**			
Firmness	−0.866	−0.925	−0.935
**C**			
Firmness	−1	−0.866	−0.859
**C_1_**			
Firmness	−0.993	−0.997	−0.658
**X**			
Firmness	−0.786	−0.866	−0.914
**X_1_**			
Firmness	−0.999	−0.999	−0.756

* Control—uncoated loaf; Loaf coated with: P—pectin; C—CMC; X—xanthan; index 1 indicates coatings in which mallow extract (*M. sylvestris*) has been added.

**Table 3:** Firming kinetic parameters during storage of loaves according to the Avrami equation (*n* = 1).

Type of Loaf *	F_o,_ g/cm^2^	F_max (t = 3),_ g/cm^2^	Avrami Equation	Rate Constant (*k*), Day^−1^	R^2^	Time Constant (1/k), Days
Control	1.10 ± 0.10 ^a^	1.87 ± 0.55 ^a^	y = −0.5280x − 0.1905	0.5280	0.9426	1.89
P	0.45 ± 0.10 ^e^	1.00 ± 0.10 ^c^	y = −0.3031x − 0.5516	0.3031	0.9347	3.30
P_1_	0.50 ± 0.10 ^d^	0.83 ± 0.12 ^d^	y = −0.3466x − 1.0575	0.3466	0.9594	2.89
C	0.58 ± 0.06 ^bc^	0.67 ± 0.21 ^f^	y = −0.4777x − 2.3739	0.4777	0.9365	2.09
C_1_	0.53 ± 0.12 ^c^	0.77 ± 0.06 ^e^	y = −0.4237x − 1.5006	0.4237	0.9668	2.36
X	0.60 ± 0.10 ^b^	1.37 ± 0.15 ^b^	y = −0.1815x − 0.2355	0.1815	0.9235	5.51
X_1_	0.50 ± 0.10 ^d^	1.07 ± 0.23 ^c^	y = −0.2177x − 0.5372	0.2177	0.9432	4.59

* Control—uncoated loaf; Loaf coated with: P—pectin; C—CMC; X—xanthan; index 1 indicates coatings in which mallow extract (*M. sylvestris*) has been added. Fo—firmness at time 0; Fmax—firmness at time 3 days. a–f: Different letters in the same column indicate statistically significant differences (*p* ≤ 0.05), according to ANOVA (one-way) and the Tukey test.

**Table 4:** Parameters of the CIELab system for the crust color of small loaf during storage.

Parameter	Storage Time, Days	Type of Loaf *						
		C	C_1_	X	X_1_	P	P_1_	Control
L*	1	54.41 ± 3.03 ^b^	52.14 ± 0.17 ^c^	50.22 ± 3.09 ^e^	51.83 ± 1.75 ^d^	56.62 ± 2.84 ^a^	55.32 ± 1.84 ^a^	55.19 ± 4.42 ^a^
	2	55.81 ± 3.05 ^a^	51.71 ± 1.71 ^b^	49.75 ± 3.67 ^c^	50.61 ± 0.48 ^b^	54.26 ± 0.76 ^a^	55.42 ± 2.30 ^a^	50.00 ± 2.94 ^b^
	3	54.98 ± 4.28 ^b^	51.43 ± 0.66 ^d^	50.83 ± 5.47 ^de^	53.24 ± 2.05 ^c^	53.30 ± 0.53 ^c^	56.56 ± 1.61 ^a^	49.22 ± 1.67 ^e^
a*	1	18.06 ± 0.93 ^b^	19.60 ± 0.22 ^a^	19.97 ± 0.61 ^a^	18.10 ± 0.32 ^b^	18.33 ± 1.27 ^b^	18.25 ± 1.34 ^b^	18.66 ± 1.52 ^ab^
	2	16.63 ± 1.10 ^c^	19.68 ± 0.11 ^a^	19.00 ± 0.29 ^a^	18.94 ± 0.40 ^a^	19.03 ± 0.45 ^a^	16.82 ± 1.34 ^c^	18.31 ± 3.20 ^ab^
	3	17.49 ± 1.57 ^c^	18.99 ± 0.67 ^b^	17.98 ± 1.09 ^c^	18.16 ± 0.69 ^b^	18.68 ± 0.55 ^b^	17.52 ± 1.97 ^c^	20.70 ± 0.28 ^a^
b*	1	32.84 ± 0.76 ^a^	32.96 ± 0.47 ^a^	32.63 ± 0.93 ^a^	31.13 ± 0.86 ^a^	33.22 ± 2.13 ^b^	32.64 ± 1.17 ^a^	32.69 ± 0.62 ^a^
	2	30.91 ± 3.43 ^b^	32.93 ± 0.71 ^a^	31.77 ± 1.66 ^a^	30.90 ± 0.85 ^b^	32.72 ± 0.22 ^a^	30.69 ± 1.47 ^b^	31.40 ± 2.07 ^a^
	3	32.30 ± 1.57 ^a^	31.37 ± 1.02 ^b^	30.78 ± 0.94 ^b^	30.46 ± 0.58 ^b^	31.66 ± 0.85 ^ab^	32.95 ± 1.72 ^a^	33.34 ± 1.00 ^a^
C*	1	37.48 ± 0.95 ^a^	38.35 ± 0.51 ^a^	38.26 ± 0.53 ^a^	36.01 ± 0.78 ^b^	37.95 ± 2.14 ^a^	37.40 ± 1.67 ^a^	37.66 ± 0.26 ^a^
	2	35.10 ± 3.51 ^b^	38.37 ± 0.57 ^a^	37.03 ± 1.45 ^a^	36.25 ± 0.88 ^b^	37.85 ± 0.32 ^a^	35.01 ± 1.67 ^b^	36.38 ± 3.32 ^b^
	3	36.75 ± 1.61 ^b^	36.67 ± 1.20 ^b^	35.67 ± 0.28 ^b^	35.47 ± 0.16 ^c^	36.76 ± 1.01 ^b^	37.32 ± 2.42 ^b^	39.25 ± 0.99 ^a^
h*	1	61.20 ± 1.10 ^a^	59.26 ± 0.11 ^b^	58.52 ± 1.45 ^b^	59.81 ± 0.75 ^b^	61.10 ± 1.86 ^a^	60.81 ± 0.97 ^a^	60.29 ± 2.47 ^a^
	2	61.64 ± 1.35 ^a^	59.14 ± 0.66 ^a^	59.09 ± 1.32 ^a^	58.49 ± 0.49 ^b^	59.34 ± 0.64 ^a^	61.28 ± 1.77 ^a^	59.93 ± 3.03 ^a^
	3	61.57 ± 2.40 ^a^	58.81 ± 0.35 ^b^	59.70 ± 2.28 ^a^	59.19 ± 1.44 ^a^	59.47 ± 0.07 ^a^	62.06 ± 1.53 ^a^	58.16 ± 0.45 ^b^
∆E	1	1.00	3.20	5.14	3.75	1.56	0.43	-
	2	6.07	2.67	0.82	1.01	4.52	5.67	-
	3	6.68	3.42	4.07	5.56	4.85	8.01	-

* Control—uncoated loaf; Loaf coated with: P—pectin; C—CMC; X—xanthan; index 1 indicates coatings in which mallow extract (*M. sylvestris*) has been added. Different letters in the same line indicate statistically significant differences (*p* ≤ 0.05), according to ANOVA (one-way) and the Tukey test.

**Table 5:** Parameters of the CIELab system for the crumb color of small loaf during storage.

Parameter	Storage Time, Days	Type of Loaf *						
		C	C_1_	X	X_1_	P	P_1_	Control
L*	1	63.04 ± 1.23 ^c^	66.05 ± 2.11 ^b^	70.62 ± 3.60 ^a^	63.01 ± 1.66 ^c^	66.25 ± 5.57 ^b^	63.10 ± 0.38 ^c^	66.13 ± 0.27 ^b^
	2	66.81 ± 1.28 ^ab^	63.59 ± 0.36 ^c^	68.25 ± 3.10 ^a^	64.88 ± 1.35 ^b^	63.44 ± 1.44 ^c^	66.99 ± 4.80 ^ab^	68.17 ± 2.51 ^a^
	3	66.16 ± 3.26 ^a^	64.77 ± 1.37 ^a^	65.24 ± 3.92 ^a^	66.05 ± 2.85 ^a^	66.97 ± 1.40 ^a^	65.41 ± 1.52 ^a^	67.36 ± 2.14 ^a^
a*	1	1.71 ± 0.04 ^b^	2.12 ± 0.50 ^a^	2.24 ± 0.17 ^a^	2.03 ± 0.49 ^a^	1.86 ± 0.07 ^b^	1.70 ± 0.13 ^b^	2.07 ± 0.26 ^a^
	2	2.10 ± 0.61 ^a^	1.70 ± 0.24 ^b^	2.27 ± 0.37 ^a^	2.30 ± 0.20 ^a^	1.98 ± 0.10 ^a^	1.71 ± 0.05 ^b^	1.91 ± 0.38 ^a^
	3	1.96 ± 0.17 ^a^	1.89 ± 0.12 ^a^	2.31 ± 0.30 ^a^	2.15 ± 0.15 ^a^	1.97 ± 0.08 ^a^	1.78 ± 0.11 ^b^	2.20 ± 0.20 ^a^
b*	1	12.99 ± 0.46 ^b^	13.93 ± 1.10 ^b^	15.22 ± 1.92 ^a^	14.45 ± 1.82 ^a^	13.75 ± 0.16 ^b^	13.87 ± 0.23 ^b^	13.83 ± 1.25 ^b^
	2	13.90 ± 0.67 ^a^	13.59 ± 0.33 ^a^	14.73 ± 0.29 ^a^	14.94 ± 0.56 ^a^	14.07 ± 0.60 ^a^	13.64 ± 0.74 ^a^	14.09 ± 0.49 ^a^
	3	13.38 ± 1.12 ^b^	13.97 ± 0.44 ^a^	15.37 ± 0.42 ^a^	14.58 ± 0.56 ^a^	13.78 ± 0.36 ^a^	13.14 ± 0.68 ^a^	14.79 ± 0.92 ^a^
C*	1	13.09 ± 0.47 ^b^	14.10 ± 1.16 ^a^	15.38 ± 1.92 ^a^	14.60 ± 1.85 ^a^	13.88 ± 0.16 ^a^	13.98 ± 0.23 ^a^	13.99 ± 1.28 ^a^
	2	14.06 ± 0.76 ^a^	13.70 ± 0.31 ^b^	14.91 ± 0.32 ^a^	15.12 ± 0.56 ^a^	14.21 ± 0.60 ^a^	13.75 ± 0.74 ^b^	14.22 ± 0.52 ^a^
	3	13.52 ± 1.27 ^b^	14.10 ± 0.44 ^a^	15.54 ± 0.37 ^a^	14.74 ± 0.57 ^a^	13.92 ± 0.35 ^a^	13.29 ± 0.68 ^b^	14.95 ± 0.90 ^a^
h*	1	82.93 ± 0.55 ^a^	81.40 ± 1.36 ^a^	81.57 ± 0.81 ^a^	82.03 ± 1.34 ^a^	82.32 ± 0.29 ^a^	82.99 ± 0.55 ^a^	81.51 ± 0.34 ^a^
	2	81.49 ± 2.01 ^a^	82.88 ± 1.11 ^a^	81.24 ± 1.31 ^b^	81.26 ± 0.65 ^b^	81.98 ± 0.08 ^a^	82.83 ± 0.23 ^a^	82.29 ± 1.36 ^b^
	3	81.65 ± 0.07 ^a^	82.27 ± 0.50 ^a^	81.42 ± 1.31 ^c^	81.62 ± 0.49 ^a^	81.84 ± 0.54 ^a^	82.31 ± 0.35 ^a^	81.51 ± 1.08 ^a^
∆E	1	3.22	0.14	4.70	3.18	0.26	3.05	-
	2	1.39	4.61	0.74	3.42	4.73	1.28	-
	3	1.87	2.73	2.20	1.33	1.11	2.59	-

* Control—uncoated loaf; Loaf coated with: P—pectin; C—CMC; X—xanthan; index 1 indicates coatings in which mallow extract (*M. sylvestris*) has been added. Different letters in the same line indicate statistically significant differences (*p* ≤ 0.05), according to ANOVA (one-way) and the Tukey test.

**Table 6:** Microbiological characteristics of the uncoated (control) and coated small loaves during storage for three days.

Storage Time, [Day]	Type of Loaf ^1^	Total Plate Count, [CFU/g] ^2^	Coliforms, [CFU/g]	Coagulase-Positive Staphylococci, [CFU/g]	*Salmonella* spp. in 25 g	Molds and Yeasts, [CFU/g]
1	Control	0	ND ^3^	ND	ND	0
	P	0	ND	ND	ND	0
	P_1_	0	ND	ND	ND	0
	C	0	ND	ND	ND	0
	C_1_	0	ND	ND	ND	0
	X	0	ND	ND	ND	0
	X_1_	0	ND	ND	ND	0
2	Control	3.1 × 10^2^	ND^3^	ND	ND	1.1 × 10^2^
	P	0	ND	ND	ND	0
	P_1_	0	ND	ND	ND	0
	C	0	ND	ND	ND	0
	C_1_	0	ND	ND	ND	0
	X	0	ND	ND	ND	0
	X_1_	0	ND	ND	ND	0
3	Control	5.8 × 10^4^	ND^3^	ND	ND	4.9 × 10^3^
	P	0	ND	ND	ND	0
	P_1_	0	ND	ND	ND	0
	C	2.4 × 10^2^	ND	ND	ND	0
	C_1_	0	ND	ND	ND	0
	X	3.3 × 10^2^	ND	ND	ND	0
	X_1_	0	ND	ND	ND	0

^1^ Control—uncoated loaf; Loaf coated with: P—pectin; C—CMC; X—xanthan; index 1 indicates coatings in which mallow extract (*M. sylvestris*) has been added.; ^2^ CFU/g—Colony Forming Units per gram; ^3^ ND = Not detected.

## Data Availability

The data presented in this study are available on request from the corresponding author.
